# Lessons from the COVID-19 pandemic for substance misuse services: findings from a peer-led study

**DOI:** 10.1186/s12954-022-00713-6

**Published:** 2022-12-12

**Authors:** Katy Holloway, Shannon Murray, Marian Buhociu, Alisha Arthur, Rondine Molinaro, Sian Chicken, Elwyn Thomas, Sam Courtney, Alan Spencer, Rachel Wood, Ryan Rees, Stephen Walder, Jessica Stait

**Affiliations:** University of South Wales, Caerleon, Wales

## Abstract

**Background:**

The measures implemented to contain the spread of the COVID-19 virus disrupted the provision of substance misuse treatment and support. However, little is known about the impact of this disruption on individuals seeking treatment for drug- and/or alcohol-related problems (henceforth service users). This study aimed to help substance misuse services learn lessons and identify ways of optimising delivery and minimising harm in the event of any future lockdowns or global crises.

**Methods:**

The study was co-produced by a team of peer researchers, practitioners, policymakers and academics. Telephone interviews were conducted with 202 substance misuse service users over a 6-month period commencing June 2020. The interviews were conducted by a small group of seven peer researchers each with lived experience of substance use problems. The interview data were recorded by the peers in an anonymous online questionnaire survey and analysed using standard quantitative and qualitative methods.

**Results:**

Service users responded to the COVID-19 pandemic in a variety of ways. Diverse responses were noted in relation to their substance use patterns, their personal lives and their substance misuse treatment experiences. For some, the pandemic acted as a new risk environment factor that increased their vulnerability to substance-related harm. For others, it facilitated aspects of the enabling environment, thereby reducing the risk of harm.

**Conclusions:**

Service users are not a homogenous group, and an individualised approach to treatment that recognises the potential for varied responses to the same stimuli is needed. The findings suggest that service users would benefit from having a choice in how they access treatment and from greater access to outreach programmes that take treatments and harm reduction tools such as naloxone into the community. The research also supports the involvement of people with lived experience in substance use research, policy and practice.

## Introduction

Since the announcement of the COVID-19 pandemic and the subsequent lockdown that followed in March 2020, there have been unprecedented changes in society. Globally and within the UK, many people have faced financial instability, social isolation, anxiety and uncertainty. Public health measures implemented to limit the spread of the virus resulted in many community-based support services being significantly impacted and, in some cases, closed. These changes often impacted those most at risk to not only the virus itself but those vulnerable to abuse, drug and/or alcohol relapse, mental health problems, homelessness and other problems [1–10].

In this paper, we focus on one of the most vulnerable and marginalised groups in society, namely people with drug and/or alcohol problems. We present findings from a peer-led, co-produced research project that sought to capture the voices and learn about the experiences of people with substance use problems at the height of the pandemic (substance use problems refers to the use of alcohol and/or drugs in amounts or by methods which are harmful to the individual or others). The primary aim is to help substance misuse services identify ways of optimising delivery and minimising drug and alcohol-related harm in the event of any future lockdowns or new global crises.

### The impact of the COVID-19 pandemic on drug and alcohol use

For many, the lockdown and measures implemented to contain the spread of the COVID-19 virus resulted in changes in drug and alcohol use and associated behaviours. Globally, cross-sectional surveys identified increases in the consumption of some substances (e.g. alcohol, cannabis, tramadol and benzodiazepines) and decreases in the use of others (e.g. MDMA and cocaine). Increases were linked to feelings of stress, fear, isolation and boredom and were more common among dependent users. Decreases were linked to the closure of pubs, bars and nightclubs where ‘recreational’ drugs are often consumed and were more common among occasional users [5, 11–15].

In response to the pandemic, treatment services for people with drug and/or alcohol problems were disrupted. While some were forced to close their doors others remained open and rapidly adapted to the ‘extraordinarily challenging circumstances’ by developing innovative methods of delivery and implementing new forms of treatment ([16], p.239). There is some evidence to suggest that the pandemic led to a decline in the number of people seeking treatment for substance use problems [16]. However, the picture is mixed as some services reported increases in referrals while others reported decreases [11, 17–20].

To identify studies that had investigated the pandemic experiences of people engaged with substance misuse treatment services (henceforth ‘service users’), we conducted systematic searches of online databases and searches of the grey literature. In total, eight UK-based studies each with a different focus, methodology and sample were identified. Given the relevance to the current paper, short summaries of this eclectic group of studies are presented below.

### The impact of the COVID-19 pandemic on service users in the UK

In a survey of 149 service users, Higgins, Kelly and Campbell [17] found that nearly half (45%) had reduced their substance use during the early stages of the lockdown, while just over one-third (37%) had increased their use. Diary entries recorded by organisational leads showed an increased demand for services stemming mainly from an upturn in the number of clients relapsing. A common message noted by service users and staff was the ‘critical’ importance of keeping lines of communication open to allow service users to ‘check-in’ with staff. Interestingly, no single form of contact (i.e. face-to-face, telephone or text message) emerged as preferable.

Kesten et al. [6] conducted interviews with 28 people who injected drugs to examine their treatment experiences during the pandemic. While some changes to the treatment landscape were criticised (e.g. the shift to remote forms of contact), other changes were welcomed. The relaxation of supervised consumption rules and the provision of take-out supplies of medication were applauded as they reduced exposure to drug-using peers and gave greater autonomy to service users. Changes in drug use patterns were linked to market factors (e.g. reduced purity and supply issues) as well as to the boredom and isolation of lockdown.

Whitfield et al. [20] examined the impact of lockdown on needle and syringe programme (NSP) provision in England. The analysis showed that the number of clients, visits and needles issued all dropped substantially from mid-March 2020. Estimates suggest that the number of needles distributed per person injecting psychoactive drugs halved from fourteen needles per week in the early stages of the lockdown. The authors conclude that more research is needed to improve understanding of the risks and harms associated with the decline in NSP use and recommend that alternative forms of provision are ‘urgently needed’ (p.3).

Seddon et al. [19] conducted interviews with 30 older adults to explore their pandemic alcohol treatment experiences. Increases and decreases in alcohol use were reported depending on a variety of factors including their physical and mental health, living arrangements and family support. In contrast to the findings reported by Higgins et al. [17], face-to-face provision was deemed ‘essential’ by older service users who experienced difficulties in accessing online support and engaging with online technology. The authors conclude that services must consider the needs of older adults in any future lockdowns to ensure that they are not marginalised by a remote model of service provision.

Parkes et al. [7, 8] conducted qualitative interviews with service users, staff and other professionals to examine how a homeless service in Scotland adapted to the pandemic and provided support to clients with substance use problems. The study found that while the pandemic created many challenges it also helped to remove barriers, particularly in relation to the provision of harm reduction services such as naloxone. The study identified mixed views on the shift to distanced communication, but the authors concluded that telephone and online provision of support was both feasible and acceptable for service users.

Schofield et al. [21] conducted semi-structured interviews with 29 people who use drugs to explore the impact of the pandemic on treatment and recovery services in Scotland. Interviewees described mixed feelings and experiences. Some described experiencing benefits from changes in policy and practice triggered by the pandemic. As also noted by Kesten et al. [6] interviewees welcomed the improvements in access to clinical prescribing and valued being trusted to take home and manage doses in larger amounts. However, interviewees also reported difficulties in accessing recovery systems due to isolation and problems in accessing general healthcare services. This was felt to be particularly problematic given that many people who use drugs have chronic physical and mental health conditions.

Smith et al. [22] investigated the experiences of seven people in recovery from problem substance use and their perceptions of the pandemic. Through repeat interviews conducted during and after the first lockdown, the study found that the pandemic experiences were mixed. While some reported boredom, loneliness and relapse events others described being unaffected by the pandemic largely because they were well used to living in a state of anxiety, isolation and uncertainty. The study highlights the varied impact of the pandemic on recovery experiences and emphasises ‘the complex and individualised role that social connectedness plays in relapse occurrence’ (p.1).

Adams et al. [23] conducted qualitative telephone interviews with 26 people who had experienced homelessness during the first lockdown period. The study highlighted barriers to accessing support that existed prior to the pandemic (e.g. limited opening hours) suggesting a need to improve service provision irrespective of any global crisis. More positively, some service users reported that the pandemic was the first time that services had worked together to support them. Others, who had been previously frustrated by a negative treatment environment (with a high presence of drugs) finally felt comfortable accessing support by telephone rather than in person. Drawing on feedback from those with lived experience of the issues, the authors recommend the need for further research to understand the perspectives of those providing support and for co-developing solutions that are acceptable to service users and providers.

### The impact of the COVID-19 pandemic on service users in other countries

Research has emerged from other countries that reflects many of the findings observed in the UK. A recurring theme is the profound disruption to services with many citing reduced access to harm reduction services, withdrawal management and treatment [24]. Although swift adaptions by service providers were made (i.e. changes to remote support and telehealth such as online-based services), many people encountered a diminished ability to mitigate the risk associated with not only the pandemic but their substance use [25–33].

Overall, the lockdown and measures implemented to contain the virus have had a negative impact on many people who use drugs and alcohol. However, substantial gaps in knowledge remain and there is a dearth of detailed information on the relative risks of COVID-19 and associated lockdowns for people with substance use problems. Given the emerging threat from new variants of COVID-19 and other diseases (e.g. Mpox) and the possibility of future lockdowns, it is critical that this gap in knowledge is filled. Service providers need to know how best to act to ensure harm is reduced and lives are saved and the most useful informants are those with lived experience of coping with substance use problems during the pandemic.

## Methods

### Context

The idea for the project emerged early in April 2020 shortly after the first lockdown had been introduced in the UK. At that time, it was believed that the pandemic presented a particular threat to people with substance misuse problems [34]. Not only was this vulnerable group thought to be at an increased risk of COVID-19 infection, it was also believed to be at an elevated risk of other drug-related harms [35]. There were particular concerns about the impact of lockdown on the use of key harm reduction methods (e.g. being able to use drugs in sight of others—see Holloway et al. [36]) and on service users’ ability to access treatment and engage with their networks of support [37–39].

In response to these concerns, we consulted with our networks of partners including treatment providers and recovery groups from across Wales and together agreed that research was needed to investigate not only the impact of the pandemic on people with drug and alcohol problems, but also the impact on substance misuse services and the people working within them. The goal was to learn lessons from the lockdown to optimise service delivery in the event of any future lockdowns or global crises. Data collection commenced in the summer of 2020 and continued for a 6-month period.

To facilitate timely and successful completion, the project was divided into three parts, each led by a different organisation but supported and assisted by the other consortium members. One strand, led by staff in Kaleidoscope (a third-sector treatment provider operating services across Wales and in the Wirral), focussed on people with substance use problems who were in contact with services. A second strand, led by staff in Barod (another third-sector provider operating services across South, Mid and West Wales) focussed on frontline staff working in treatment services. The third strand, led by researchers in the Substance Use Research Group (SURG) at the University of South Wales (USW), focussed on the views and experiences of decision-makers (e.g. senior managers, Chief Executives, public health officials, service commissioners, and policymakers).

The project was successful in generating three rich data sets [40–42]. In this article, we present findings from the first strand of the research focussing on the experiences of people with substance use problems during the pandemic and associated lockdowns of 2020. We plan to publish findings from the other two strands of research in separate journal articles.

### Research design

The research was based on a cross-sectional design, which involved the collection of data from a subset of the population of interest at a point in time [43]. A mixed strategy was used that involved the collection of quantitative and qualitative data using an online questionnaire survey [44]. The questionnaire examined the impact of COVID-19 on various aspects of service users’ lives, including their: use of substances, use of harm reduction methods, access to clinical/prescribing services, mental and physical health, families and relationships, finances and employment, access to support networks and access to direct and crisis support from treatment services. The questionnaire included closed questions where respondents were asked either to select their answers from preset lists of responses or to rate their views on Likert scales. The questionnaire also included open questions that allowed respondents to provide more detailed responses.

The questionnaires were completed during telephone interviews with service users. These ‘survey interviews’ were conducted by a small team of seven peer researchers each with lived experience of substance misuse problems. The decision to use a peer-led approach was influenced by the success of a peer-led ‘secret shopper’ exercise undertaken in needle exchange schemes in South Wales in 2014. Not only did that research yield authentic and reliable data, but it also provided the peer researchers with a lasting sense of purpose and value [42]. Engaging people with lived experience in policy, practice and research (sometimes referred to as Patient and Public Involvement or PPI), is becoming increasingly important across the UK [45–47]. Our approach therefore aligned neatly with guidance on service user involvement in research and is a good example of research that has been co-produced by people with lived experience, in partnership with practitioners, policymakers and academics.

In practice, our team of peer researchers was more heavily involved in certain parts of the research process than in others. However, they made important contributions at each stage and their involvement was more substantive than the ‘pockets of co-production’ described in other co-created studies ([48], p. 6).

### Recruitment of peers

The peer researchers were recruited from all seven Welsh regions through the consortium’s extensive networks. Upon recruitment, the peers were brought together for a bespoke online training session that lasted several hours and covered a range of topics including safeguarding, confidentiality, conduct and boundaries, consent, data entry, interviewing tips, jargon busting, and the use of incentives. In subsequent online discussions and through email exchanges, the peers developed the survey questionnaire using a combination of their own questions amalgamated with those from other key stakeholders.

Each peer was provided with a telephone, preloaded with sufficient credit, that could be used to conduct the interviews. The peers supported one another throughout the project via a WhatsApp group chat and through weekly debriefing Zoom calls with Kaleidoscope staff. At the end of the project, a meeting was held during which the peers were able to share and discuss their research experiences.

### Data collection

Service users were approached by their key workers and asked if they would be interested in taking part in a ‘survey interview’ with a peer researcher. For those who agreed, a ‘consent to contact’ form was signed by their key worker and emailed to the peer along with the service user’s name and telephone number. The peers subsequently rang the number and after confirming consent to participate, completed the interview. At the end of the interview, respondents who wished to enter the free prize draw (to win shopping vouchers) were asked to provide their contact details, which were recorded on a separate piece of paper and stored in a sealed box. Winners were drawn randomly at the end of the survey at which time all materials were collected from the peers and either shredded or digitally destroyed.

The interview process was not always straightforward. In practice, some service users could not be reached on the phone numbers they had given. Some answered the phone but asked for a call back later, which added to the peers’ workloads. Nevertheless, despite these difficulties, in-depth interviews were conducted with 202 service users. On average, the interviews lasted about an hour. In one case, the service user was so keen to talk to someone that the interview lasted for 90 min. The positive impact of the peer researchers on the interviewees was noted in other cases too. For example, one key worker contacted Kaleidoscope staff to let them know how well one of the peers had responded to a service user who had been struggling with their mental health.

### Analysis

Anonymised survey responses were entered into the online survey (hosted on Survey Monkey) either during the course of the interview or at the end, depending on the peers’ multitasking and technological capabilities. The final survey data set was downloaded and imported into SPSS Version 28 for cleaning, coding and analysis by USW researchers. Quantitative data were analysed using simple frequency counts and, where appropriate, cross-tabulations and nonparametric statistical tests. Qualitative responses were coded into key themes in new variables leaving the original responses available for inclusion as ‘*evidence*’ of key themes. A grounded theory approach was used to code the data which involved reflecting on the existing (fairly limited) evidence base and identifying new and emerging themes from our own analyses [49]. The coding process was completed by two members of the research team (KH, SM) and checked for consistency and accuracy by one other (MB).

It is important to note that while the peers worked hard to capture direct quotations from service users during the interviews, this was not always possible. The ‘*evidence*’ presented in this paper must therefore be viewed as a hybrid of verbatim quotations (written in the first person) and paraphrased summaries (often written in the third person). In line with good practice in the reporting of qualitative research, the lengthier ‘*responses*’ are attributed to specific service users by way of code numbers [50]. The ‘*responses*’ are included to help add vividness to the content and also to give service users a voice in the research outputs. It is hoped that the quotations will also help readers to assess the accuracy of the analysis and thereby strengthen the findings [51]

### Ethics

Ethical approval was obtained from USW for SURG researchers (a) to conduct secondary analyses of data collected through our consortium partners, and (b) to conduct empirical research with senior managers and decision-makers. In the knowledge that articles would be written based on the secondary analysis of data collected through our consortium partners, formal guidance was provided to them to ensure that the data were collected in compliance with the USW and British Society of Criminology ethical codes of practice.

### Sample characteristics

The sample was fairly evenly split in terms of gender with equal numbers of men and women and a small number of gender fluid, non-binary, other and people with undisclosed gender taking part (see Table 1). The inclusion of so many women is interesting and unexpected given that women are under-represented in treatment services and also in drugs research [52–54]. We asked the peers for their thoughts on this and they offered two possible explanations: (1) the peer-led approach made women feel more comfortable in disclosing their experiences, and/or (2) the novel impact of the pandemic, which research suggests impacted more heavily on women than men, made some women feel that it was important to share their views [55].

**Table 1:** Characteristics of the sample

	Frequency	%
Male	95	47
Female	95	47
Other	7	6
18–34	58	29
35 +	139	71
White British	162	80
White English	14	7
Mixed—White and Black Caribbean	3	2
Mixed—White and Black African	1	< 1
Mixed—White and Asian	1	< 1
Black—Caribbean	1	< 1
Black—African	1	< 1
Black—Indian	1	< 1
Other ethnic groups	13	6
Total	202	100

Some missing cases

While no gender differences were noted, differences were noted in terms of the age and ethnic group composition of the sample (see Table 1). In relation to age, the sample was more heavily weighted in favour of older people (aged over 35) with nearly three-quarters of the sample falling into this age group. In terms of ethnic group, the vast majority of respondents were either White British or White English with only a minority of respondents from minority ethnic groups. Unlike gender, the composition of the interview sample in terms of age and ethnic group reflects that of the population engaged in substance misuse treatment in Wales [56, 57].

Among those who provided information about their ‘primary’ substance (i.e. the substance that caused them the most issues for which they were currently receiving support), alcohol was the most commonly reported substance either singularly (63%, *n* = 108) or in combination with other substances (5%, *n* = 8). When drugs were identified as a primary substance, this was more commonly heroin/opiates (12%, *n* = 20), cannabis (5%, *n* = 8), or cocaine (4%, *n* = 6).

The sample was fairly mixed in terms of the type of treatment and support that they were receiving at the time of the interview (see Table 2). Among those who provided the information, more than half indicated that they were receiving psychosocial treatment either alone or in combination with other kinds of support. The sample was also mixed in terms of the length of time that they had been receiving this treatment (see Table 2). Among those who provided the information, more than three-quarters had been in treatment for less than 5 years, while just over one-tenth had been in treatment for 5 years or more.

**Table 2:** Current treatment and support

Type of treatment	Frequency	%
Psychosocial	97	48
Psychosocial +	32	16
OS alone	10	5
Employment/training alone	10	5
Clinical support	15	7
Unknown	38	19
*Length of time in treatment*		
< 1 year	79	39
1–4 years	75	37
10 + years	16	8
5–9 years	11	5
Unknown	21	10
Total	202	100

## Results

The ‘survey interviews’ generated a wealth of data relating to the impact of COVID-19 on service users’ lives. To illustrate the breadth and depth of impact felt by our participants, we present findings covering a wide range of issues. For clarity, these issues are grouped into three broad themes, namely the impact that the pandemic had on their: (1) drug and alcohol use, (2) personal lives and (3) access to treatment and support. Where we present the results of quantitative analyses, we focus on valid cases only (i.e. we exclude any missing cases). Italicised text denotes extracts from service users’ survey interview responses.

### Drug and alcohol use

A key aim of the research was to examine the impact of the pandemic and lockdown on drug and alcohol use patterns and related behaviours. Interviewees were therefore asked a series of questions probing whether there had been changes in the frequency of their substance use, the quantities consumed, and the financial costs involved. While most service users either maintained their pre-pandemic patterns of use or decreased them during the lockdown period, a significant minority reported increases (see Table 3). For drug use, increases were reported by roughly one-third of service users. For alcohol use, the increases were slightly larger with more than two-fifths reporting increases in frequency and more than one-third reporting an increase in quantity consumed (see Table 3).

**Table 3:** Impact of the pandemic on drug and alcohol use behaviours

	Decreased	Same	Increased	Total
Frequency drug use	35% (20)	31% (18)	35% (20)	100% (58)
Frequency alcohol use	34% (35)	23% (24)	43% (45)	100% (104)
Quantity drug use	30% (17)	34% (19)	36% (20)	100% (56)
Quantity alcohol use	33% (35)	29% (30)	38% (40)	100% (105)
Cost drug use	28% (15)	36% (19)	36% (19)	100% (53)
Cost alcohol use	31% (32)	38% (39)	32% (33)	100% (104)

Those service users who had used drugs prior to the pandemic were asked to comment on whether they changed the type of drug(s) that they used. Most respondents (73%, *n* = 38) indicated that there had been no changes during the lockdown period. The 14 (27%) service users who reported changes described some interesting differences. Some reported using drugs that they had not used previously.

Experimenting with new substances. (197)

Took MDMA once which they don't usually. (155)

I started smoking crack. (69)

I have started using spice which I never did before. (145)

I’ve used cannabis once that I wasn’t before. (56)

In some cases, the new substance was used as a substitute for another substance. Indeed, one service user described increasing their use of alcohol specifically to reduce their drug use. By contrast, another service user described using drugs specifically to reduce their alcohol use.

Drank more alcohol rather than taken drugs during lockdown. (29)

Smoking cannabis to cut out the alcohol. The urge to drink is lessened by smoking cannabis. (144)

In one case, however, it was clear that new substances were being used in addition to and ‘*on top*’ of substances used prior to the pandemic:

I am now using crack cocaine and street Valium, on top of alcohol. (3)

Perhaps unsurprisingly given that most people had not changed the type of substances that they used, most respondents reported that their method of administration had also remained unchanged. When changes were reported (*n* = 7), these reflected the changes in the type of drug being used (e.g. a shift from drinking alcohol to smoking cannabis). However, one service user did report that they had ‘*started to inject again in lockdown*’ and another reported that they were now smoking as well as injecting.

#### Drug supply and purchasing

At the start of the pandemic, there were concerns about the impact of the lockdown on the drug supply network [38, 39]. Questions were therefore included in the interview probing whether service users had experienced any difficulties in accessing their usual supply of drugs and/or whether their usual purchasing patterns had changed during lockdown. In relation to the former, two-thirds of interviewees (66%, *n* = 40) reported that access had remained the same and in a small number of cases access had reportedly become less difficult (8%, *n* = 5). However, in just over one-quarter of cases (26%, *n* = 16), service users reported that accessing drugs had become more difficult.

When asked about changes to their purchasing pattern, the majority (78%, *n* = 38) reported that it had remained the same during lockdown. Those who did report changes (22%, *n* = 11) were asked to describe the nature of those changes. The responses were varied and included shifts to potentially less harmful behaviours such as *‘increased purchasing through friends’* and ‘*taking only what is prescribed*’ by a healthcare professional. However, shifts to potentially more harmful behaviours were also reported. This included purchasing drugs more frequently (‘*I now score where I can’, ‘I had to go out more to help my tolerance’*), purchasing larger quantities (*‘bought stock’, ‘buying and using more and buying larger quantities’*) and purchasing drugs personally rather than through friends (‘*Never bought drugs for myself before’).*

#### Lapse/relapse

Research has shown that overcoming drug or alcohol dependence takes time and that experiencing a lapse or relapse is a natural part of the process [58]. Those service users who had maintained a period of abstinence during the pandemic were asked if they had lapsed or relapsed at any point. In one-quarter of cases, interviewees reported that they had. When asked for further details, interviewees provided sad stories that clearly linked their lapse/relapse to ‘*feeling depressed*’, ‘*worry and loneliness*’, and the ‘*boredom*’ associated with lockdown. For one service user, her relapse was the result of ‘*peer pressure*’ from her partner who was ‘*drinking heavily*’ while for another it was the isolation and loss of formal coping mechanisms:

I was abstinent for 2 years 7 months prior to lockdown. I maintained my sobriety until roughly July which by that point I had been self-isolating since March/April. My coping mechanisms such as peer support groups, voluntary work, daily exercise, daily contact with my parents were all halted, leaving me at a loss, bored, agitated and angry. (38)

### Personal lives

An important part of the interview was to consider the impact of the pandemic on different aspects of service users’ lives. The interview therefore included questions relating to harm reduction behaviours (both COVID-19-related and substance-related), finance and employment, family and relationships, and physical and mental health.

#### Harm reduction—COVID-19 related

In the early days of the pandemic, concerns were raised about the likelihood of service users complying with social distancing rules, using hand sanitiser, and wearing personal protective equipment (PPE) [59]. The vast majority of service users reported that they had received clear messages and information about keeping safe during the pandemic (85%, *n* = 150) and most either followed or did their best to follow the guidance (92%, *n* = 161). Contrary to such stigmatising expectations, the vast majority of service users said that they had used hand sanitiser and PPE during lockdown (93%, *n* = 162) and more than half reported changing the way they behaved in certain social situations (54%, *n* = 86).

When asked to explain how their behaviour had changed, the service users indicated that they had become ‘*more spatially aware*’, ‘*more wary*’ and ‘*more anxious*’ of others, particularly of ‘*anyone coughing*’. Many explained that they tried to keep their distance and avoid contact wherever possible (e.g. ‘*avoiding handshakes*’, ‘*100% no sharing with anyone*’, ‘*can no longer hug people*’). One service user commented on the importance of social distancing as a mark of respect for others:

Maintaining social distancing when in public. Being more respectful to other people's space. (77)

In some cases, interviewees described going out less often than before, ‘*meeting in much smaller groups*’ and making changes to their alcohol use and purchasing behaviours:

No longer drinking alcohol in groups, drinking more alone at home. (62)

No dealer, but got alcohol with takeaway more rather than supermarket/corner shop. (24)

Don't go out unless absolutely necessary. (79)

#### Finance and employment

Across the UK, periods of lockdown designed to halt the spread of COVID-19 had serious financial consequences for many people [60]. For service users, the problem was particularly acute with more than one-third (37%, *n* = 67) reporting financial problems during the pandemic and roughly one-quarter (24%, *n* = 44) reporting employment-related problems.

For some, the lockdown resulted in an increase in expenditure on drugs and/or alcohol, which drained their financial resources (e.g. ‘*we spent our money on drugs*’, ‘*increased alcohol use*’, ‘*increased use of heroin*’). One service user attributed the increase in expenditure to the need to buy her ‘*son his alcohol*’.

Others suffered financially through a range of employment-related issues, including ‘*lost employment*’, ‘*lost clients*’, ‘*being unable to work due to illness*’, ‘*getting furlough pay*’, ‘*no furlough available’*, ‘*job hours changed*’ and ‘*no overtime work available*’. One service user reported that he had been unable to sell the Big Issue during lockdown while another had been unable to work for 4 months:

My career has centred on hotels and catering, was shut down for 4 months … (90)

Was selling the Big Issue so had to stop during lockdown. (12)

Those without jobs prior to the lockdown also suffered financially during the pandemic. The reasons were varied and included: not being ‘*able to make much money begging*’, ‘*less jobs to apply for*’, job contracts being ‘*ended*’ or ‘*put on hold*’ and service users being ‘*unable to attend job interviews due to their cancellation*’. Problems with benefits were also reported:

Lost my PIP, my ESA was cut down, then couldn't afford to insure my car so lost that with my independence, fell into debt with phone bill. (43)

Several interviewees commented on the loss of volunteering opportunities during lockdown, which for one service user had a negative effect on their mental health*.*

A range of other financial burdens were also reported, including the costs of caring for family members during the pandemic (e.g. ‘*son was made redundant so I had to support*’), increased household costs due to more time being spent at home, and costs associated with behaviours designed to minimise the risk of catching COVID-19:

Lodger had to move out due to risk, no income from their rent. (75)

Have to use taxis, anxious to go out. (99)

Had to shop at local spar and this was more expensive. (23)

A small number of interviewees explained that the financial implications of lockdown resulted in them needing to access the foodbank:

Used more electric and gas. Had to go to food bank for all the food needed. (112)

Money was reduced but I had foodbank vouchers from [drug service] (72)

However, the story was not wholly negative as a small number of interviewees described positive financial and employment outcomes. Some interviewees suggested that they were ‘*better off*’ and had ‘*improved finances*’ during the pandemic while others reported job improvements or new careers:

In a good way—led me back to caring profession when I was doing retail and very unhappy in that job. (77)

Got a new job which I like. (60)

#### Families and children

COVID-19 had a profound impact on families across the world [61] and our interviewees were no exception. Nearly two-thirds (64%, *n* = 116) reported that the pandemic had negatively impacted their family in some way. The main problems reported were linked to having ‘*no access*’ or ‘*not being able to see*’ and spend time with family members, particularly children. Sadly, several reported that they had lost family members during the pandemic:

I lost my dad to covid, can’t see family at the moment. (60)

Mother died just before Christmas, suspected Covid-19, been a very tough time. (114)

A wide range of other family-related problems were also reported and included issues related to ‘*financial worry*’ and the consequences of spending either too much time together (e.g. ‘*arguments*’) or too much time alone (e.g. ‘*loneliness’, ‘isolation became horrible’*). One service user explained this well:

Being in each other’s company 24/7, you can get on top of each other. Relationships have become strained at times. (140)

For some, the family difficulties had serious consequences including the ‘*break up’* of relationships and relapse into substance use:

Family strain was a contributing factor to me relapse. (161)

Close proximity exacerbated family relationships—being coupled up caused rows and I think was an influencing factor in my relapse. (175)

While many people described experiencing family problems during the pandemic, a significant minority (43%, *n* = 77) indicated that there had been some positive consequences too. Some benefited from ‘*spending more time*’ and ‘*closer contact*’ with family members, which resulted in a ‘*much better bond*’ and ‘*strengthened*’ relationships with children and parents. Others described reconnecting with friends and family (e.g. ‘*got contact with my family again’, ‘opportunity to connect with people/friends from afar’*) and developing new relationships (e.g. ‘*met a new girlfriend!’).* Others, however, benefited from the time and space away from other people that lockdown afforded (e.g. ‘*Time away from my sister’, ‘Has helped keep influencers away’).*

Some respondents reported that lockdown had given them time to reflect on their substance misuse problems and enabled them to ‘*realise how far I have come’*, and how their past behaviours had impacted their family members:

Allowed time to reflect on situation with family and previous substances use. (17)

The time for reflection led some to be more honest with family members. One service user described how they had ‘*finally*’ reached out and asked for help while others spoke of the positive consequences of being honest with their family about their use or abstinence:

I have been more honest with my mam and sisters about my cocaine use and they are now supporting me more. (10)

Maintained sobriety during lockdown and reported that to family which was positive. (63)

#### Physical health

Respondents were asked during the interview whether lockdown had impacted their physical health in any way. In particular, they were asked to indicate whether lockdown had impacted their diet, exercise, sleep, teeth and caring responsibilities (see Table 3). Problems with sleep (40%, *n* = 81) and exercise (44%, *n* = 88) were the most commonly reported physical health issues and a few interviewees described a combination of several health problems:

Care responsibilities: my mother had a stroke, so I was also caring for her. All have altered due to lockdown, in particular sleep patterns. (114)

No exercise, sleep terrible, put on weight. (124)

I suffer with chronic fatigue syndrome so access to exercise has gone down as has my sleep quality. (116)

Health problems associated with diet and nutrition were also common and were reported by about one-third of the sample. Weight gain was reported by many interviewees and this was linked in several cases to the lockdown restrictions that prevented them from meeting their ‘*walking group’, ‘going to the gym’*, and limited how far they could cycle. In short, the ‘*rules made exercise difficult*’.

While negative health issues were widely reported, some interviewees described a range of positive health impacts. For some, lockdown encouraged healthier choices in exercise and diet. Service users described losing weight ‘*due to gardening’, ‘eating properly and exercising*’ and were ‘*feeling better*’ for doing so. Others described how they had ‘*gotten fitter during lockdown*’ and how their ‘*health was improved by walking more and less snacks*’.

When asked if they felt that substance misuse services could have done more to help them with their physical health, financial/employment or family problems, most service users felt that nothing more could have been done. However, a couple of interviewees suggested that more outdoor activities would have been useful.

#### Mental health

Like many other people around the world, many of the service users in our sample suffered from mental health problems as a direct result of the pandemic. Indeed, nearly three-quarters (73%, *n* = 132) of the sample indicated that their mental health had been impacted during lockdown. During the interviews, many discussed the long periods of isolation and limited contact with anyone as a cause of their declining mental health.

Not getting out socialising has impacted on my mental health. (150)

We’re social creatures and it’s not natural to be on your own. (157)

Got down and anxious and just not right when haven't been able to see or speak to anyone for a few days. (125)

Being locked in sets her off, gets bored, then angry and things escalate from there. (96)

For some, the lockdown exacerbated existing conditions:

Anxiety has been worse and having to stay in has increased depression. (135)

Suffer with panic attacks and depression. Alone in the flat all of the time, not able to go out. Felt isolated and took more of my medication. (83)

Not being able to engage in routine activities was identified as a source of anxiety for several respondents:

No gym had a massive effect on me lots of anxiety and stress. (56)

Not being able to do my activities like volunteering etc. really affected me and made me so much more isolated and depressed. (113)

Many felt that the decline in peer support groups within the services contributed to their mental health problems. In some cases, the lack of support groups played a key role in triggering a relapse and slippage back ‘*into bad habits*’:

My mental health issues increased dramatically during lockdown. Being unable to attend weekly peer support groups face to face and continuing with my voluntary roles meant a large portion of my distraction/coping mechanisms were inaccessible to me. (38)

Just a lack of support generally to group meetings and this ended. Lonely and boredom at times leading to relapse. (59)

Some service users described becoming so mentally unwell during lockdown that they became suicidal (e.g. *‘wanted to go and end it’, ‘suicidal thoughts have come back the past week’*). When asked to comment about the nature of their mental health problems during lockdown, it was not uncommon for respondents to refer to the helpful support provided by substance misuse services but a lack of support from other organisations:

Mental health services have been no help, [drug service] have been fantastic regarding mental health. (138)

Only support I had was off [drug service]—I would have ended it if it wasn’t for my worker. (83)

Regular contact with keyworker has been very beneficial to mental health, particularly when other services have not been so supportive, such as GP. (78)

I couldn’t access my psychologist and could only access psychiatrist through phone calls but there was no extra support, my support worker couldn’t have done more—she’s brilliant. (175)

One service user commented on the ‘*massive failings in mental health support*’ and suggested that there should have been ‘*far more energy’* put into third-sector services. In particular, it was felt that more advertising and more outreach work (possibly even ‘*door to door*’ distribution of information) was needed to direct people to appropriate help and support. It was felt that improved signposting would be useful for both those who had not sought help before as well as those who had engaged with support groups previously:

Better signposting to Peer Support Groups–the local meetings moved online and individual signposting to a specific meeting would have been very helpful to maintain existing links with known peers (in person) with strong recoveries. (80)

Many service users felt that the continued provision of ‘*face-to-face appointments*’ during lockdown periods would be beneficial to service users. It was also suggested that a regular ‘*weekly phone call to check in*’ could have provided support and helped service users cope with the loneliness and boredom during times of isolation. Others stated that services should attempt to ‘*be more available and open during lockdown*’ and that there should be ‘*more involvement with mental health services*’. The benefits of maintaining contact were widely recognised and one service user suggested that this might be facilitated through *‘a newsletter sent out each week with updates, quizzes *etc.*’*.

### Access to treatment and support

#### Maintaining contact with services

The vast majority (95%, *n* = 173) of service users reported that they had maintained contact with their key workers during the pandemic and had continued to receive ongoing support. In nearly two-thirds of cases, the frequency of contact remained the same, but in just over a third of cases the frequency was noted to have changed. The sample was fairly evenly split in terms of whether the change was an increase or decrease in frequency.

#### Type of support provided

Just under two-thirds of interviewees (65%, *n* = 116) stated that the nature of the support changed during the pandemic, most notably through a shift from face-to-face contact to telephone contact (71%, *n* = 82). About one-fifth of service users (21%, *n* = 24) reported that they had received online support, and in most cases this involved video calls. While a small number of interviewees indicated a preference for telephone meetings (14%, *n* = 28) or a flexible approach (14%, *n* = 28) depending on various factors such as their levels of anxiety, the vast majority indicated a preference for face-to-face interaction (70%, *n* = 127). The main reasons given were because face-to-face meetings were more ‘*personal*’ and ‘*social*’, allowed for non-verbal communication and helped counter feelings of isolation and loneliness:

I like the social aspect and being able to leave the house. (50)

I feel it offers more of a personal feel thus giving the service user more of a feeling of empathy and belonging among peers. (38)

There were more silences, can’t see what thinking (104)

Face to face I would look forward to … a reason to get up and go out. (83)

Those who reported a preference for telephone support explained that this was because they suffered from ‘*anxiety when meeting people*’, it was more flexible in terms of timing and avoided the financial costs of travelling to appointments:

Childcare for children, it’s easier on the phone. (114)

Phone—I find travelling hard as I am in a rural area. (101)

I work full time shifts away from where I live and can’t take time out of work very easily. (10)

#### Crisis support

During lockdown, nearly two-fifths of interviewees (39%, *n* = 72) reported that they had required crisis (i.e. urgent) support from their key worker. The most pressing reasons for this were: family/relationship issues, mental health problems, their medication, housing and benefits. The other reported issues included Social Services referral, police involvement, hospital admissions, the need for food, drug/alcohol relapse and a combination of two or more problems.

Some service users felt that they were unable to request help due to mental health issues or because they did not want to access online support: *“I cannot request help when I’m in a bad place,” “I needed help but felt it would be pointless doing it over the phone.”* However, most felt supported and were able to access their key workers/support staff when they needed to do so:

I needed a listening ear due to some family issues, and my worker was always there. (138)

Just knowing my support worker was there made my crisis easier to manage. (157)

#### Clinical and prescribing services

In response to social distancing rules and concerns over the transmission of COVID-19 during lockdown, important changes were made to clinical services. To reduce the risk of catching the virus and of infecting others, some service users were given take-out supplies of prescription medication. Others were given an alternative form of long-acting medication (i.e. Buvidal) that required less frequent attendance at the service or pharmacy.

When asked if they had been able to access clinical services during lockdown, more than half (*n* = 70, 56%) said that they had not. The reasons for this are not wholly clear as few explanations were provided. This means that important information about the experience of receiving clinical services during the pandemic is unknown. However, those who did provide reasons referred to delays in accessing prescriptions and some logistical difficulties. One service user explained that their prescription had been ‘*sent across town without notification*’, which meant they were ‘*unable to collect it*’. Another described ‘*problems getting Antabuse*’ while another reported that their local service had ‘*closed*’.

A small number of respondents explained that while they had been able to access clinical services, it had not been straightforward but over time and with help, the situation had improved:

Prescriptions didn’t turn up but keyworker sorted it out for. (126)

Bit awkward initially with new rules and hours, but fine afterwards. (125)

Difficulty in accessing methadone from chemist in a different town, changed to weekly. (136)

More positively, respondents reported few difficulties in accessing their medication from community pharmacies. Indeed, the vast majority (*n* = 91, 90%) reported that they had not any issues or problems.

Those who were given larger take-out doses of medication during lockdown were asked how this made them feel. The responses were mixed with some referring to positive feelings, some referring to negative feelings and others referring to a status quo. Positive responses included feeling ‘*much more empowered*’ to take control of their health and ‘trusted’ to not misuse their medication. This made service users feel ‘*good*’ and one described being ‘*more determined not to mess it up*’. Negative responses were less common and tended to be related to feelings of vulnerability or frustration that they were not able to get the medication/dose that they had wanted (e.g. ‘*was promised monthly Buvidal but was denied’, ‘not given doses of methadone which I would have liked to save me going out more’*).

#### Harm reduction

With the restrictions on social distancing and reduced access to services during lockdown, concerns were raised about the extent to which service users would be able to engage in potentially life-saving harm reduction practices (e.g. not using alone or out of sight of others) [62]. An open-ended question was therefore asked that probed (a) whether interviewees had received any specific harm reduction messages from services during lockdown, and (b) whether they had found that advice was helpful. While most of those who answered the question indicated that they had not received any such advice, a large minority (41%, *n* = 35) said that they had. This included ‘*being issued with Prenoxad*’ (a form of naloxone, which reverses an opioid overdose), general harm reduction advice (e.g. *‘overdose info’*), and more specific advice on safer alcohol consumption (e.g. ‘*drinking water between drinking’, ‘gradual reduction from alcohol, changing strength, *etc.*’*). One service user commented that their ‘*worker always talks about harm reduction during appointments*’.

Naloxone is an important harm reduction tool, which helps to prevent drug-related deaths [63]. Relatedly, during the lockdown period, nearly one in ten interviewees reported that they had either experienced or witnessed a non-fatal overdose (9%, *n* = 13). When probed for further details, it emerged that in several cases, the overdose was intentional:

Try to overdose using Quetiapine and Lorazepam. (93)

Tried to overdose in an attempt on my life. (51)

Took an overdose of Diazepam when I ran out of my medication, I took them all at once. (83)

In one case the interviewee explained that a friend had been given naloxone by another friend and had survived:

Friend overdosed at a funeral was given Prenoxad by another friend and survived. (27)

Given the life-saving properties, it is noteworthy that few interviewees reported accessing naloxone during lockdown. Indeed, only 14 of the service users interviewed in this study indicated that they had obtained naloxone.

## Discussion

This paper has presented findings from a co-produced, peer-led research project that investigated the impact of the COVID-19 pandemic on people with substance misuse problems. Drawing on qualitative ‘survey interviews’ with a sample of 202 substance misuse service users, the study examined the impact of the pandemic on their drug and alcohol use, their personal lives, and their substance misuse treatment experiences.

While there is a growing body of research investigating the impact of the COVID-19 pandemic on substance misuse and related behaviours, our literature searches identified only a limited amount of research based on a peer-led approach involving people living in the UK. The work that we did find included research undertaken by the International Network of People Who Use Drugs [64], which involved peers from across 50 countries, but only one wholly UK-based study [23]. This was not entirely unexpected given that in substance use research generally, few studies have embodied the principles of co-production and relatively little co-produced research has been published [45]. Our study therefore makes an important contribution to ‘an emerging field’ that recognises (a) the right of ‘patients’ (or people with lived/living experience of substance misuse problems) to be part of research aimed at their wellbeing, and (b) the critical value of their insights and experiences [45].

The peer-led approach was beneficial in many respects including the recruitment of a large sample of service users. The peer researchers reported improvements in their self-esteem and self-confidence as a result of their involvement and service users appeared to benefit from the opportunity to discuss their problems with someone with lived experience of similar challenges. Furthermore, collaborating with substance misuse services facilitated the early and wide dissemination of findings through an infographic-style online report (and subsequently a webinar), which translated the results into visual, easily understood information suitable for a wide audience [40, 65].

However, our peer-led approach was not without its limitations. As novice researchers, the peers were unfamiliar with the demands of empirical research, which may have contributed to inconsistencies in data-recording practices and more missing data than we had anticipated. These limitations might be considered a small price to pay for the many benefits that co-production brings. Indeed, the ‘dark side’ of co-production is to some extent inevitable and has been described as part of the ‘rub’ of ‘managing the conflicting demands of empirical research with effective co-production methodologies’ [66], p.1., [67] p.56).

While the main aim of this study was to ‘learn lessons’ for substance misuse services for any future lockdowns or global crises, the study also provided an opportunity to learn lessons for the co-production of substance use research. Our experiences suggest that it would be helpful to offer more intensive training to peer researchers and to thoroughly pilot both the data collection process and the analysis. The co-creation of research outputs that meet the needs of each collaborating partner is also recommended. Journal articles are not always the best vehicle for generating impact outside of the Academy and the lengthy academic peer-review process can delay the publication of important findings [66]. Co-creating outputs in accessible formats and disseminating them through both academic and non-academic platforms could therefore help to speed up the dissemination process and maximise impact.

Consistent with studies of similar populations in the UK, the service users in this study responded to the pandemic and adapted to the lockdown environment in a variety of ways [17, 19, 21]. While some maintained the status quo making little, if any, changes to their patterns of drug and alcohol use, others changed the frequency of their use, the quantities consumed and/or the types of substance that they used. Varied responses to the pandemic were also reported in other aspects of the service users’ personal lives as well as their treatment experiences, highlighting the individualised nature of the impact of the pandemic.

The diverse range of responses reported by the service users in our study support Nordgren and Richert’s [32] assertion that people who use drugs are not a homogenous group. Rather, they are a group of individuals who respond and adapt to crises and events in different ways depending on a wide range of factors. Understanding what these factors are and how they interact to escalate or reduce the risk of harm is therefore an important goal if lessons are to be learned for the future.

Previous research suggests that vulnerability to drug-related harm is strongly associated with the social or physical spaces in which drugs are used [68, 69]. Within these spaces (or risk environments) it is argued that a variety of physical, social, economic, and policy-related factors interact at different levels to shape the drug-using environment. While some researchers have focussed on how these factors work to escalate the risk of harm [68], others have considered how they can decrease exposure to drug-related harm and shape an ‘enabling’ environment [70].

Recent research drawing on the perspective of social workers in Sweden identified the COVID-19 virus as a ‘new’ risk environment factor that increased vulnerability to drug-related harm among some people who use drugs [32]. However, the virus was also noted to facilitate aspects of the enabling environment thereby reducing the risk of harm among some members of this population. The results of our study support both of these conclusions as explained below.

First, the pandemic and associated macro-level lockdown measures impacted the institutional level by forcing services and support groups to shift away from face-to-face delivery to online and telephone formats. For some service users, this affected their ‘risk environment’ and increased their vulnerability by restricting their access to activities and support systems that had previously helped them to cope with substance misuse-related problems. For others, such as those living in rural communities and those with social anxiety problems, the shift to remote forms of delivery had an ‘enabling’, harm-reducing effect in allowing them to access support more easily, cheaply and comfortably than previously (see also [23]).

Second, the macro-level lockdown measures resulted in significant changes to the prescription and distribution of opioid substitution treatment (OST). This included the roll-out of long-acting, injectable buprenorphine (Buvidal) and the wider provision of take-out supplies of other forms of OST. For some service users, the provision of take-out supplies influenced their enabling environments through an increased sense of empowerment and responsibility (see also [6, 21]). The impact of these changes on the risk environment was less obvious. However, some service users expressed frustration at not being prescribed Buvidal while others reported difficulties and delays in accessing scripts from community pharmacies, which could have led to the onset of painful withdrawal symptoms and relapse.

Third, the macro-level lockdown measures on some occasions acted as a risk-generating factor directly on service users without the intermediary effect of institutional-level factors. Indeed, for some service users relapse incidents and problems in their personal lives were triggered by the boredom, anxiety, and loneliness of lockdown. For others, however, the lockdown measures had an enabling, harm-reducing effect by providing greater opportunities for exercise, outdoor activities and spending time with family members. In some cases, lockdown led service users to cut down their substance use.

Fourth, the lockdown measures impacted local drug markets, which in turn affected drug purchasing patterns. For some, the changes altered the ‘enabling’ environment through shifts to less harmful behaviours such as purchasing drugs through friends (hence reducing their risks of being apprehended by the police) or limiting their use to legally prescribed (unadulterated) medication. For others though, the changes affected the ‘risk’ environment as they began to purchase drugs more frequently, in larger quantities and personally rather than through friends thereby increasing their risk of arrest, robbery and increased use.

Finally, Welsh Government’s harm reduction approach has for many years helped to shape an enabling environment through the distribution of naloxone (a life-saving, overdose-reversing drug) to people at risk of an opioid overdose. Our findings highlight that this enabling environment was negatively affected by the policies imposed during the pandemic. Due to the social distancing restrictions and the lockdown measures, only a small number of service users reported accessing naloxone during lockdown. This meant that important opportunities for harm reduction were lost and that service users (and those around them) were at an increased risk of a fatal overdose (see also [20]).

## Conclusion

In line with other pandemic-focussed research, our study has highlighted the varied ways in which service users responded to the pandemic. While lockdown created problems and increased the risk of harm for some people, it created opportunities and reduced risks for others. Clearly, service users are not a homogenous group and an individualised approach to treatment that recognises the potential for varied responses to the same stimuli is needed.

Other lessons for substance misuse services include the need for a hybrid system of online/face-to-face delivery that offers service users a choice in how they access support and treatment. Services are also advised to implement outreach programmes that take treatments and harm reduction tools (such as peer-to-peer naloxone distribution programmes and the ‘spike on a bike’ mobile needle exchange schemes) directly into communities. Services might also benefit from routinely reflecting on current practice and being bold enough to identify new and innovative styles and forms of treatment and proactively (rather than reactively) trial them in appropriate conditions in close collaboration with service users. Services are also encouraged to recognise the empowering potential of a more relaxed and trusting approach regarding the dispensing of OST and to resist the temptation to resume ‘normal’ pre-pandemic practices.

There are also lessons to be learned for policymakers. Indeed, our study has shown that macro-level decision-making can have a detrimental effect on ‘enabling environments’ that have long histories of success in reducing the risk of harm among vulnerable groups. It is important that measures designed to reduce societal harm do not cause more harm than they prevent. Finally, our research adds to the evidence base that supports the continued and extended involvement of people with lived experience in substance use research, policy and practice.
